# Effects of forages on the microbiota of crossed sheep on cold Plateau

**DOI:** 10.1080/10495398.2024.2362639

**Published:** 2024-06-10

**Authors:** Yue Ren, Renzeng Ciwang, Khalid Mehmood, Kun Li

**Affiliations:** a Institute of Livestock Research, Tibet Academy of Agricultural and Animal Husbandry Sciences, Lhasa, PR China; b Key Laboratory of Animal Genetics and Breeding on Tibetan Plateau, Ministry of Agriculture and Rural Affairs, Lhasa, PR China; c Faculty of Veterinary and Animal Sciences, The Islamia University of Bahawalpur, Bahawalpur, Pakistan; d Institute of Traditional Chinese Veterinary Medicine, College of Veterinary Medicine, Nanjing Agricultural University, Nanjing, PR China; e MOE Joint International Research Laboratory of Animal Health and Food Safety, College of Veterinary Medicine, Nanjing Agricultural University, Nanjing, PR China

**Keywords:** Provender, tibetan sheep, ileum, flora, sequencing

## Abstract

Diet is an important component to influence microbiota, there are less data available about the microbiome of Suffolk cross with Tibetan (SCT) animals with different fodders. The current study was conducted for comparing the fungi microbiota in SCT sheep fed with different forages. Sequencing of ileum samples from sheep groups of AH (alfalfa and oat grass), BH (mixture of grass and concentrated feeds), CH (concentrated feed I), DH (concentrated feed II) and EH (concentrated feed III) achieved 3,171,271 raw and 2,719,649 filtered sequences. Concentrated feeds changed fungi microbiota in SCT sheep with three phyla and 47 genera significantly different among the groups. Genera include positive genus of *Scytalidium* and negative fungi of *Sarocladium*, *Kazachstania*, *Gibberella*, *Scytalidium*, *Candida*, *Wickerhamomyces*. The findings of our study will contribute to efficient feeding of SCT sheep at cold plateau areas.

## Introduction

Intestine microbiota are composed of numerous microbes which include fungi, viruses, bacteria and some protozoa^1^ which are responsible for 98% of the organisms genetic activity.^2^ Gut microbiota plays a significant role in functions related to the nutrition, metabolism, immune system development, nervous system development and behaviour of the host.^3^ Fungi in gut biomes have not been studied as much as bacteria. However, it has been reported that gut fungi play an important role on health and diseases of animals^4^ as they affect intestinal bacteria through fungal–bacterial interaction.^5^ In the human gut, there are over 400 fungi species which is approximately 0.1% of the total microbial DNA in the intestine.^6^ The main fungi in the intestine include *Candida*, *Cryptococcus* and *Cladosporium* contributing greatly to the host’s immunity and gastrointestinal function.^7^^,^^8^ Studies have revealed that probiotic *Saccharomyces cerevisiae var. boulardii* is useful in treating gastroenteritis.^9^

An increase in useful gut bacteria results in better growth performance however is also related to age, diet, management and environmental factors.^10^^,^^11^ Fungi dysbiosis is commonly associated with diverse diseases like non-alcoholic fatty liver disease^12^ inflammatory bowel disease^13^ and diarrhea.^14^ Diet is another factor affecting microbiota.^15^ To date, limited information is available about gut fungi of SCT (Suffolk cross with Tibetan) sheep reared on the cold plateau with different feeding regimes. Therefore, we conducted this study to compare the fungi flora in SCT sheep fed with different forages.

## Materials and methods

### Experiment design

A total of 50 SCT sheep with average age of 3 months were equally grouped (*n* = 10) into AH, BH, CH, DH and EH. Sheep in group AH, CH, DH and EH were given alfalfa and oat grass, concentrated feed #1 (corn 35%, wheat bran 23.5%, bean pulp 10%, alfalfa 20%, highland barley straw 10.5%, premix feed 0.5% and salt 0.5%), concentrated feed #2 (corn 45%, wheat bran 11.8%, soybean oil 2.5%, bean pulp 14.3%, alfalfa 17.4%, highland barley straw 8%, premix feed 0.5% and salt 0.5%) and concentrated feed #3 (corn 50%, wheat bran 5.9%, soybean oil 3.8%, bean pulp 16.2%, alfalfa 16.3%, highland barley straw 6.8%, premix feed 0.5% and salt 0.5%), respectively. Group BH was fed with equal amount of alfalfa and oat grass, and concentrated provender I, II and III. The SCT ruminates were reared for 120 days and weighed twice daily before slaughtering to collect ileum samples.

### SCT sheep microbiota sequencing

Total DNA extraction from ileum samples of SCT sheep (*n* = 25) were carried out through MolPure® Stool DNA Kit (Yeasen, China) by following the manufacturer’s instructions. The quality and quantity of SCT sheep’s DNA extracts were checked by 1.5% agarose gel electrophoresis and NanoDrop One/OneC (Thermo Scientific, USA), respectively. Targeting gene amplification of SCT sheep products was performed by piloting ITS1 primers pairs as described previously,^16^ and then, the amplified products were again checked for quality and quantity. Final results obtained from SCT sheep were used through MiSeq reagent kit v3 (Illumina, USA), whereas sequencing was done through platform of Illlumina MiSeq in Bioyi biotechnology company (Wuhan, China).

### Fungal microbiota analysis

Amplicon sequence variants (ASVs) were generated by DADA2 after raw sequence quality control.^17^ Then, ASVs were used to align ITS database for obtaining taxonomy table through vsearch software.^18^ Differences of alpha (Shannon, observed OTUs, Faith’s phylogenetics and Chao1) and beta indexes (Qiime2β, unweighted pair-group method with arithmetic means, principal coordinate analysis and non-metric multidimensional scaling) were calculated via QIIME2.^19–22^ The distinguished marker fungi species among SCT sheep in group AH compared to other groups were analysed through analysis of variance (ANOVA), LEfSe and DEseq2.^23^^,^^24^ The KEGG Ortholog function of SCT sheep gut fungi was calculated by PICRUSt.^25^

### Statistical analysis

Analysis of variance and Duncans multiple range test was selected for obtaining significance among SCT sheep by using SPSS (IBM, 26.0). Data are presented as means ± SD and differences were perceived as statistically significant at *P* < 0.05.

## Results

### Fungal microbiota analysis of SCT ruminant with different fodders

**Figure 1.**
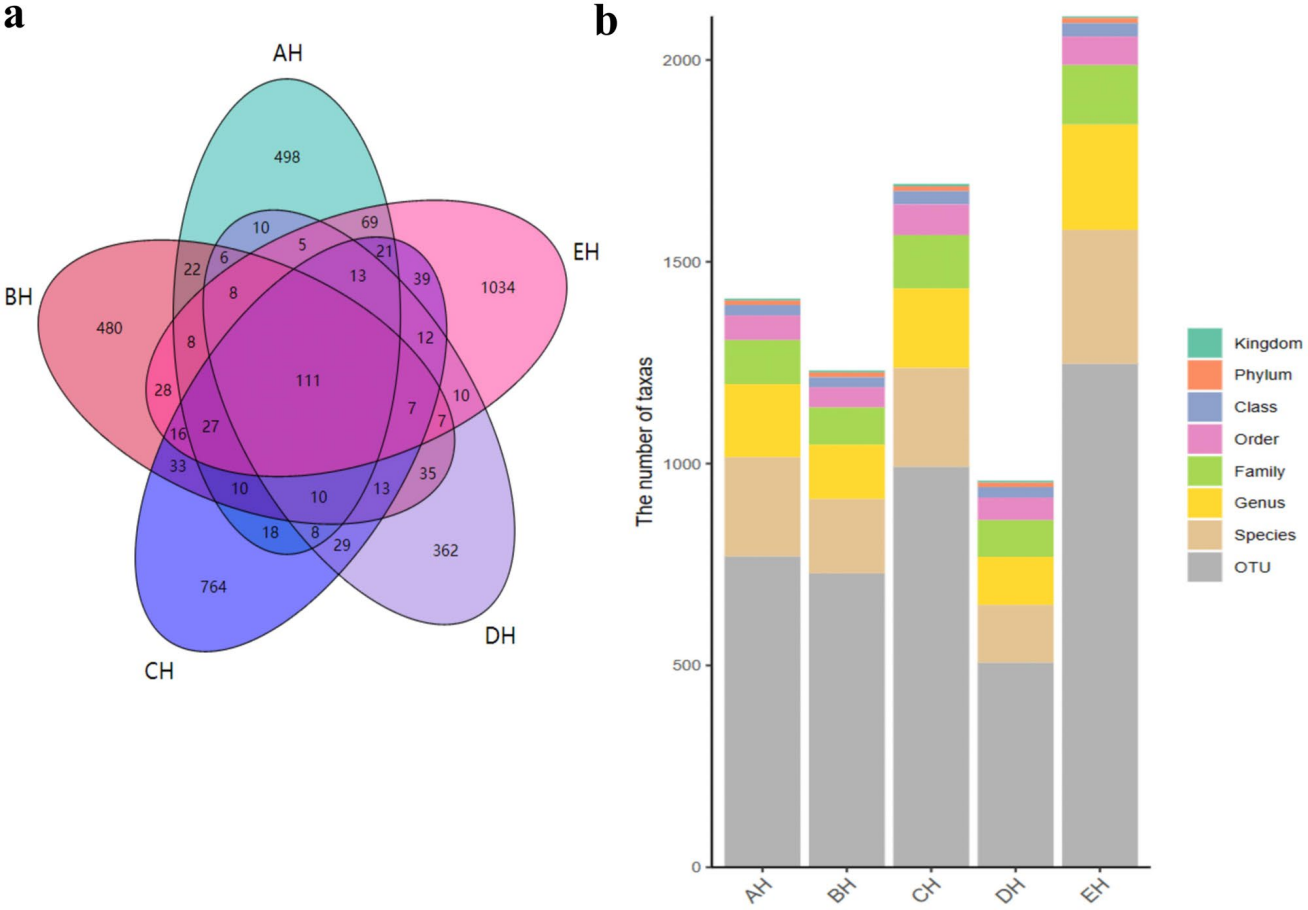
ASVs Aligning analysis. a: Venn map, b; annotation statistics.

**Figure 2.**
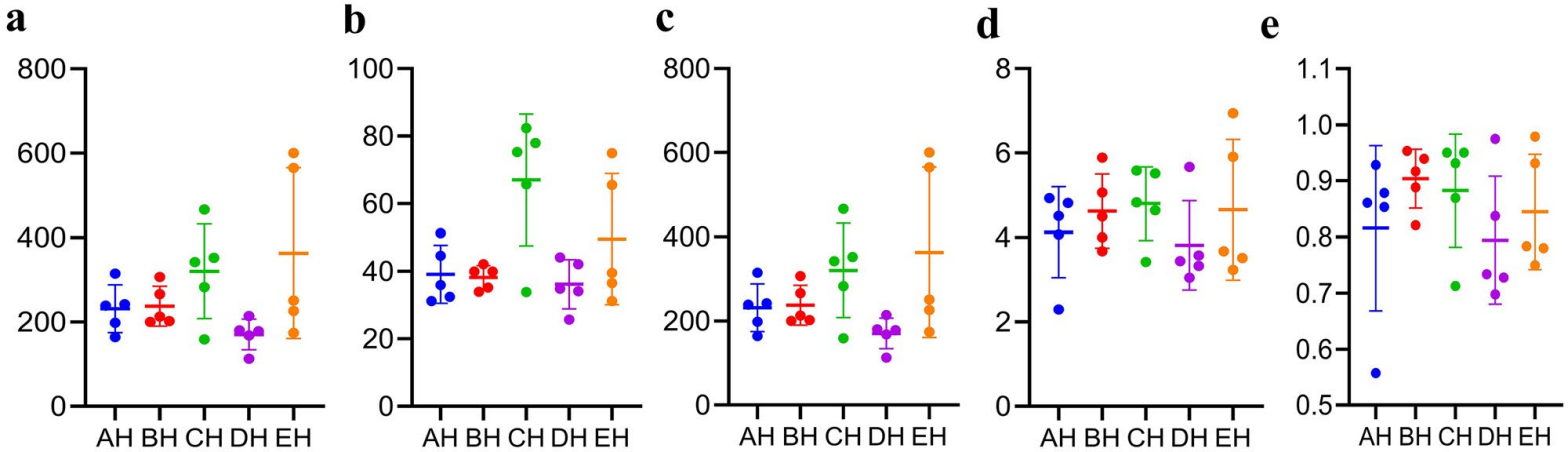
Comparing analysis of alpha diversity in SCT ruminants. a: chao1, b: faith_pd, c: observed_features, d: shannon_entropy, e: simpson.

It was observed that the average raw sequences and filtered sequences in AH (108865, 93806) were lower than BH (134633, 114510), CH (136942, 116449), DH (128162, 110202) and EH (125651, 108961) (Table 1). A total of 3249 ASVs were aligned in SCT sheep (AH = 609, BH = 591, CH = 875, DH =473 and EH = 1145) with 111 shared ASVs in small ruminants (Figure 1(a)). The shared ASVs between AH and BH, AH and CH, AH and DH, and AH and EH was 202, 208, 171 and 268, respectively. There were 11 (AH), 12 (BH), 12 (CH), 11 (DH) and 13 (EH) phyla whereas, 181 (AH), 135 (BH), 197 (CH), 119 (DH) and 261 (EH) genera observed in SCT sheep (Figure 1b()). Alpha diversity shows that no valid difference is examined among different SCT groups (Table 2 and Figure 2).

**Table 1.** The obtained sequencing data in SCT ruminants.

Sample ID	Input	Filtered	Percentage of input passed filter	Denoised	Merged	Percentage of input merged	Non-chimeric	Percentage of input non-chimeric
AH1	55105	48289	87.63	48069	45542	82.65	45236	82.09
AH2	134263	112886	84.08	112311	90767	67.6	90725	67.57
AH3	135127	120049	88.84	119418	114769	84.93	113474	83.98
AH4	114824	97972	85.32	97177	83419	72.65	83375	72.61
AH5	105006	89837	85.55	89148	83449	79.47	82472	78.54
BH1	146052	125988	86.26	125163	108481	74.28	107668	73.72
BH2	134161	114115	85.06	113379	95214	70.97	94933	70.76
BH3	124226	104652	84.24	103916	95867	77.17	94215	75.84
BH4	132954	112978	84.98	112582	96331	72.45	94130	70.8
BH5	135772	114820	84.57	114222	99815	73.52	99675	73.41
CH1	143626	120819	84.12	117371	107831	75.08	106786	74.35
CH2	134376	113414	84.4	111320	93089	69.28	92510	68.84
CH3	138205	119868	86.73	118266	111325	80.55	109837	79.47
CH4	132278	112299	84.9	110086	97561	73.75	97290	73.55
CH5	136227	115847	85.04	114856	108805	79.87	108675	79.77
DH1	135952	118146	86.9	117090	110171	81.04	107486	79.06
DH2	140623	122630	87.2	121832	104874	74.58	100071	71.16
DH3	133362	117121	87.82	116054	108824	81.6	106765	80.06
DH4	96205	78091	81.17	77523	76610	79.63	75056	78.02
DH5	134671	115023	85.41	113710	94820	70.41	93617	69.52
EH1	93775	81004	86.38	79242	74920	79.89	73186	78.04
EH2	136377	117225	85.96	116718	105483	77.35	105419	77.3
EH3	125504	109875	87.55	108326	101428	80.82	97479	77.67
EH4	136510	118234	86.61	117559	103245	75.63	101642	74.46
EH5	136090	118467	87.05	117895	108922	80.04	107337	78.87

**Table 2.** Comparing analysis of alpha diversity index in SCT ruminants.

Sample	chao1	faith_pd	observed_features	shannon_entropy	simpson
AH1	242	35.93900003	242	4.928982988	0.860615511
AH2	198	32.48047936	198	4.512180234	0.928239853
AH3	164	31.19520084	164	2.296201317	0.557217692
AH4	239	44.63034634	239	4.071550991	0.853301335
AH5	315	51.29365408	315	4.816534912	0.878159661
BH1	213	39.93466695	213	4.002531592	0.888005129
BH2	200	35.2266728	200	3.679477277	0.82063151
BH3	307	42.12849798	307	5.88764901	0.953007321
BH4	202	33.930672	202	4.49849994	0.916814954
BH5	266	39.93367113	266	5.060261448	0.939339735
CH1	352	77.98227405	352	4.645840769	0.869111106
CH2	466	82.36802327	466	5.585502367	0.950406934
CH3	283	65.73324838	283	3.425817439	0.712530706
CH4	342	75.29744116	342	5.513812339	0.950014487
CH5	159	33.85396095	159	4.830457892	0.931324553
DH1	214	44.10544252	214	3.446457763	0.733508906
DH2	180	34.08511076	180	3.329922755	0.727994892
DH3	178	42.13277823	178	3.0499653	0.697773514
DH4	113	25.73579984	113	5.673129211	0.97484255
DH5	168	34.83451306	168	3.575683199	0.837072096
EH1	600	74.91858074	600	6.945823316	0.978598107
EH2	226	36.57333991	226	3.677398128	0.780254301
EH3	565	65.51442849	565	5.91135886	0.93105107
EH4	251	39.51380359	251	3.515932827	0.783809372
EH5	174	31.19826921	174	3.235380661	0.74977987

**Figure 3.**
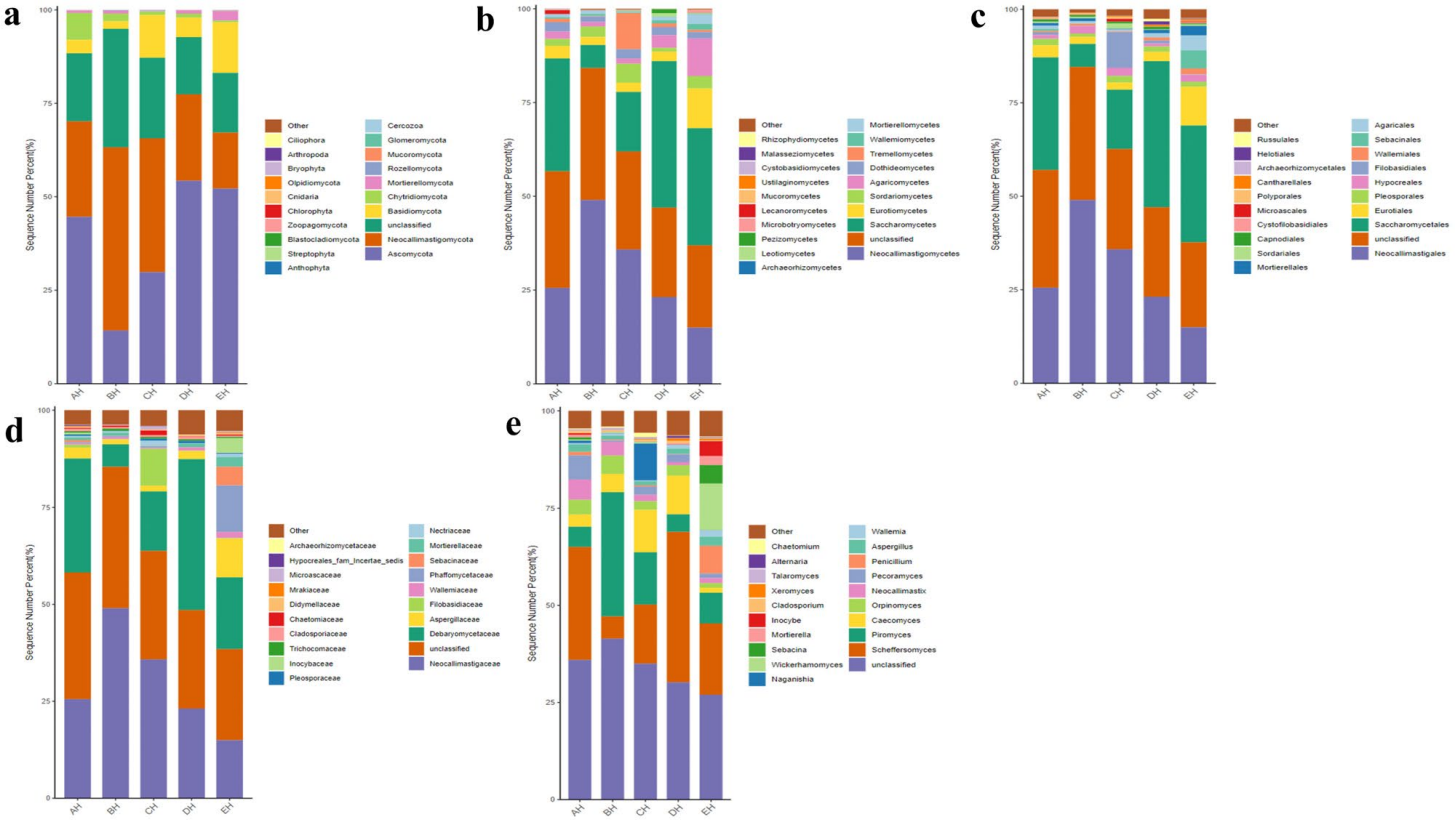
Fungi microbiota structure of SCT sheep at different taxa presented in percentile pile bar chart. a: Phylum, b: Class, c: Order, d: Family, e: Genera.

**Figure 4.**
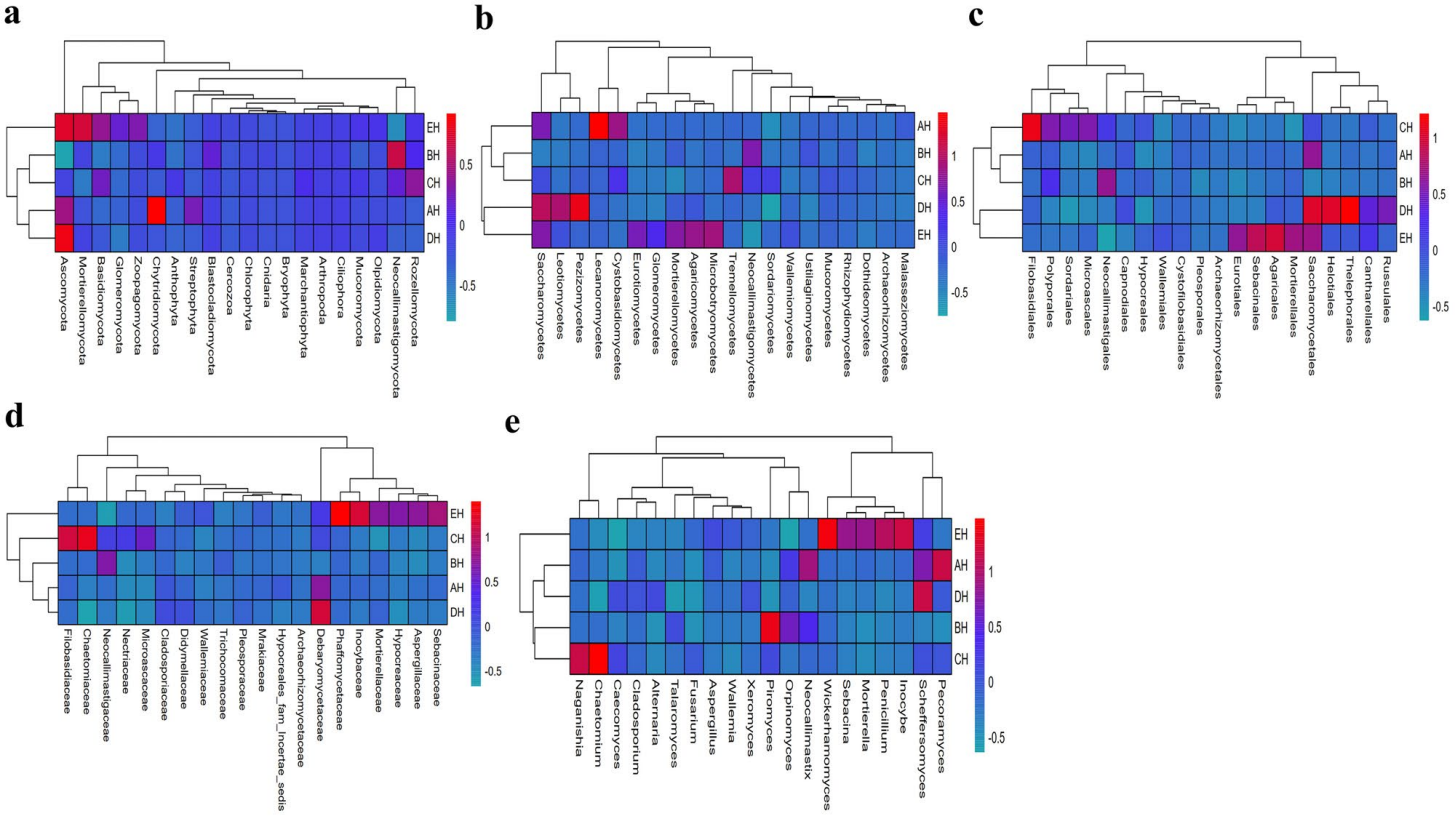
Comparing analysis of fungi microbiota structure of SCT sheep via grouping and clustering heat map. a: Phylum, b: Class, c: Order, d: Family, e: Genera.

**Figure 5.**
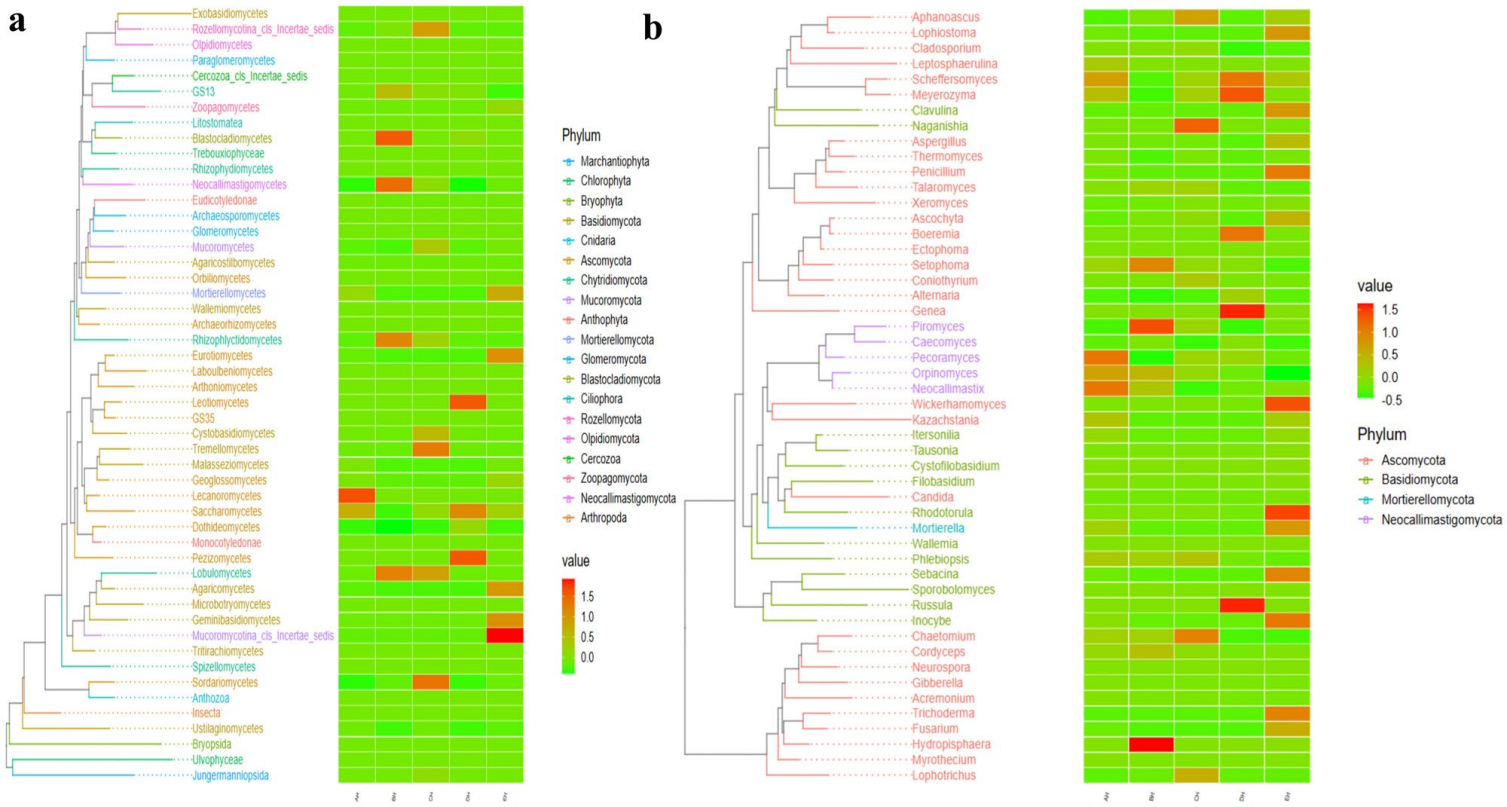
Evolutionary tree of species with heat map analysis of the fungi microbiota of SCT sheep in (a) class and (b) genera.

At the phylum level, Ascomycota, Neocallimastigomycota and Chytridiomycota were primary phyla in AH (44.64%, 25.56%, 7.14%) and BH (14.23%, 49.01%, 2.03%), whereas Ascomycota, Neocallimastigomycota and Basidiomycota were the dominating phyla in CH (29.83%, 35.81%, 11.43%), DH (54.29%, 23.12%, 5.20%) and EH (52.20%, 14.98%, 13.53%) (Figure 3(a)). At class levels, Neocallimastigomycetes, Saccharomycetes and Eurotiomycetes were mainly examined in AH (25.56%, 30.08%, 3.36%) and EH (14.98%, 31.21%, 10.59%). The principally classes in BH were Neocallimastigomycetes (49.01%), Saccharomycetes (6.12%) and Sordariomycetes (2.78%), in CH Neocallimastigomycetes (35.81%), Saccharomycetes (15.85%), Tremellomycetes (9.62%) and in DH were Neocallimastigomycetes (23.12%), Saccharomycetes 39.06%) and Agaricomycetes (3.36%), respectively (Figure 3(b)). At the order level, Neocallimastigales, Saccharomycetales and Eurotiales were the central orders in AH (25.56%, 30.08%, 3.26%), DH (23.12%, 39.06%, 2.48%) and EH (14.98%, 31.21%, 10.36%) while, Neocallimastigales (49.01%), Saccharomycetales (6.12%) and Hypocreales (2.00%) were found higher in BH and Neocallimastigales (35.81%), Saccharomycetales (15.85%) and Filobasidiales (9.53%) were prime orders in CH (Figure 3(c)). At family level, Neocallimastigaceae, Debaryomycetaceae and Aspergillaceae were mainly found in AH (25.56%, 29.39%, 2.84%), BH (49.01%, 5.77%, 1.23%) and DH (23.12%, 38.94%, 2.08%), while Neocallimastigaceae (23.12%), Debaryomycetaceae (38.94%) and Filobasidiaceae (2.08%) were the primary families in CH. Neocallimastigaceae (14.98%), Debaryomycetaceae (18.49%) and Phaffomycetaceae (12.01%) were dominating families in EH (Figure 3(d)). At genera levels, the main genus in different SCT sheep groups were *Scheffersomyces* (29.09%), *Pecoramyces* (6.21%) and *Neocallimastix* (5.22%) in AH, *Piromyces* (31.97%), *Scheffersomyces* (5.73%) and *Orpinomyces* (4.73%) in BH, *Scheffersomyces* (15.21%), *Piromyces* (13.47%) and *Caecomyces* (10.89%) in CH, *Scheffersomyces* (38.76%), *Caecomyces* (9.96%) and *Piromyces* (4.50%) in DH and *Scheffersomyces* (18.37%), Wickerhamomyces (12.00%) and *Piromyces* (7.91%) in EH, respectively (Figure 3(e)). Grouping and clustering heat map detected that Ascomycota was more abundant in AH, Neocallimastigomycota was higher in BH, Ascomycota was more abundant in DH and Ascomycota and Mortierellomycota were more abundant in EH (Figure 4(a)). At the class level, Saccharomycetes, Lecanoromycetes and Cystobasidiomycetes were more abundant in AH Neocallimastigomycetes was higher in BH, Tremellomycetes was higher in CH, Saccharomycetes, Leotiomycetes and Pezizomycetes were higher in DH, and Saccharomycetes, Eurotiomycetes, Agaricomycetes, Mortierellomycetes and Microbotryomycetes were more abundant in EH, respectively (Figure 4(b)). At the order level, higher abundance of Saccharomycetales in AH, Neocallimastigales in BH, Filobasidiales in CH, Saccharomycetales, Helotiales and Thelephorales in DH, and Saccharomycetales, Eurotiales, Sebacinales, Agaricales and Mortierellales in EH were detected, respectively, in SCT sheep groups (Figure 4(c)). Higher abundance families of Debaryomycetaceae in AH, Neocallimastigaceae in BH, Filobasidiaceae and Chaetomiaceae in CH, Debaryomycetaceae in DH and Aspergillaceae, Phaffomycetaceae, Sebacinaceae, Mortierellaceae, Inocybaceae and Hypocreaceae were revealed in EH (Figure 4(d)). At the genera level, abundance of *Scheffersomyces*, *Neocallimastix* and *Pecoramyces* in AH, *Piromyces* and *Orpinomyces* in BH, *Naganishia* and *Chaetomium* in CH, *Scheffersomyces* in DH, and *Penicillium*, *Wickerhamomyces*, *Sebacina*, *Mortierella* and *Inocybe* in EH were found (Figure 4(e)). Evolutionary tree of species with heat map analysis exhibited higher abundance of Saccharomycetes and Lecanoromycetes in AH, Lobulomycetes, Rhizophlyctidomycetes, Blastocladiomycetes and Neocallimastigomycetes in BH, Sordariomycetes, Lobulomycetes, Tremellomycetes and Rozellomycotina_cls_Incertae_sedis in CH, Pezizomycetes, Saccharomycetes and Leotiomycetes in DH and Agaricomycetes, Geminibasidiomycetes, Mucoromycotina_cls_Incertae_sedis, Eurotiomycetes and Mortierellomycetes in EH (Figure 5(a)). Higher genus of Scheffersomyces, Pecoramyces, Neocallimastix and Orpinomyces in AH, Hydropisphaera, Setophoma and Piromyces in BH, Chaetomium and Naganishia in CH, Russula, Genea, Meyerozyma, Scheffersomyces and Boeremia in DH, and Trichoderma, Sebacina, Inocybe, Clavulina, Lophiostoma, Penicillium, Mortierella, Rhodotorula and Wickerhamomyces in EH were also observed (Figure 5(b)).

### Examining the marker fungi species in SCT sheep

**Figure 6.**
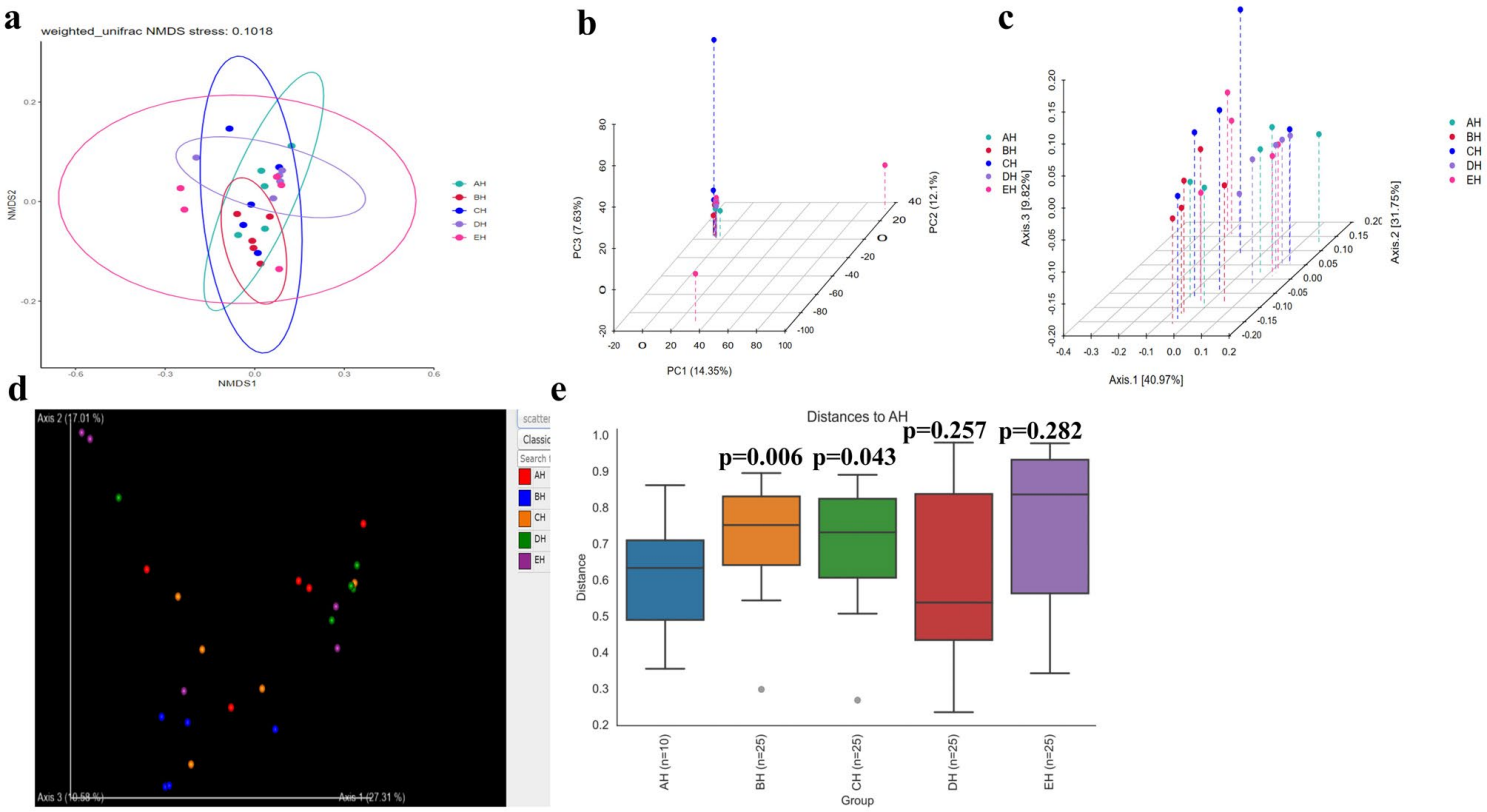
Beta diversity analysis of SCT sheep. a: NMDS, b: PCA, c: PCoA, d: Qiime2β, e: Group significance plots.

**Figure 7.**
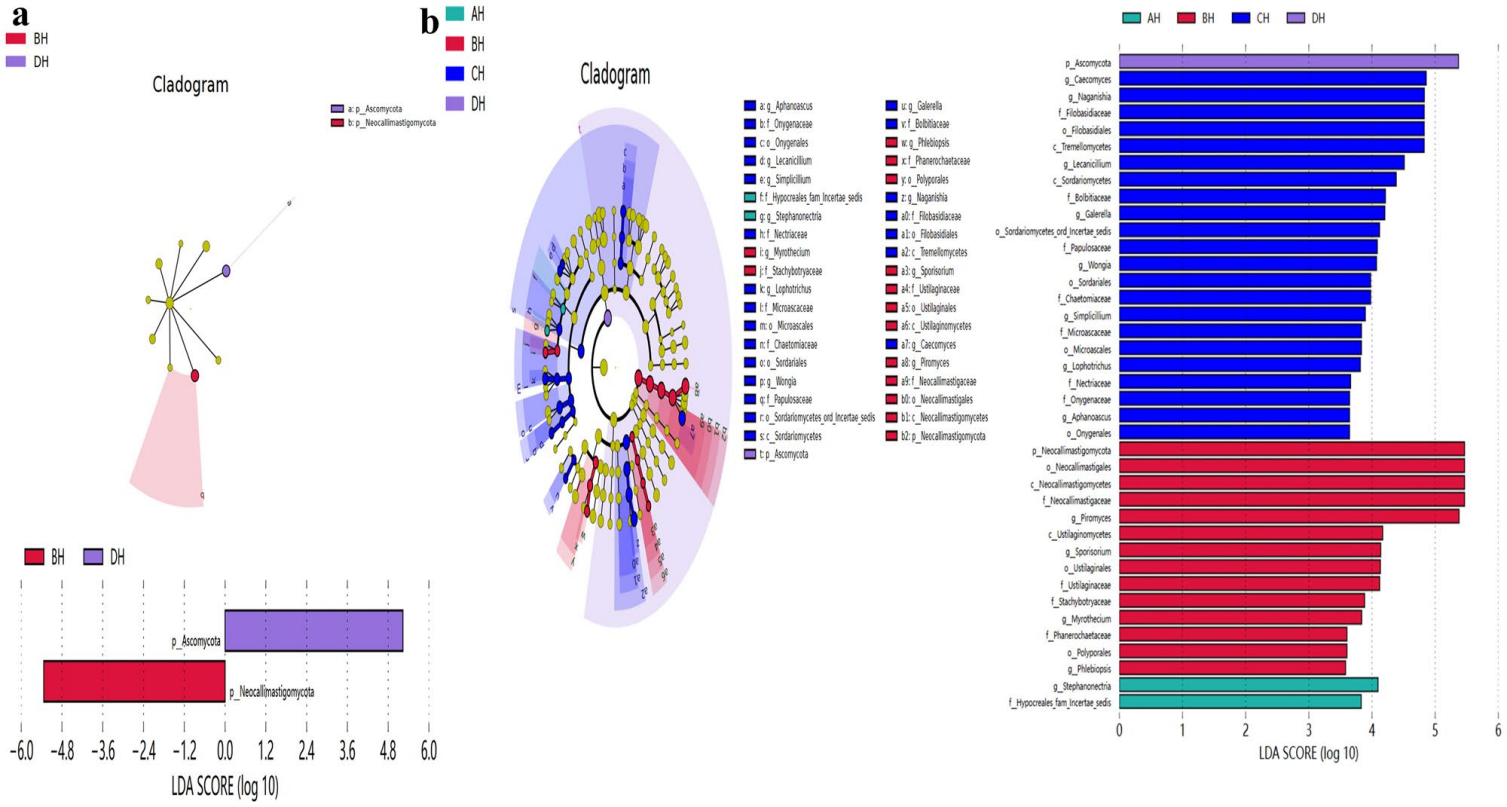
Exploring the significant different fungi species among SCT sheep via LEfSe. a: Phylum, b: Genera.

**Figure 8.**
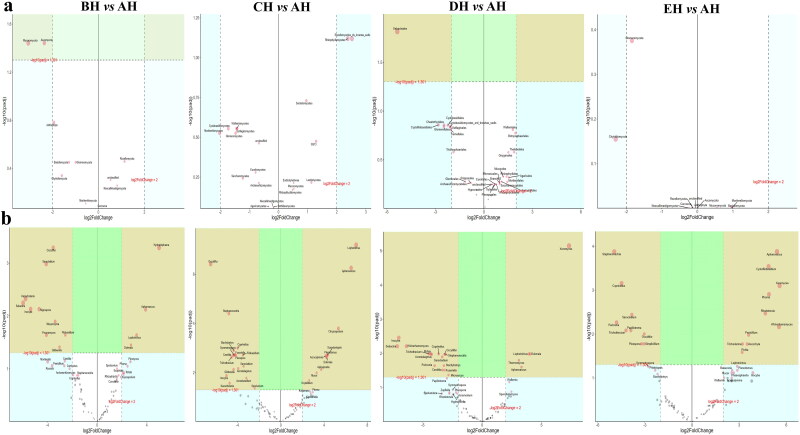
Discovering valid fungi species among SCT ruminants via DESeq2 volcano map a: Phylum, b: Genera.

**Figure 9.**
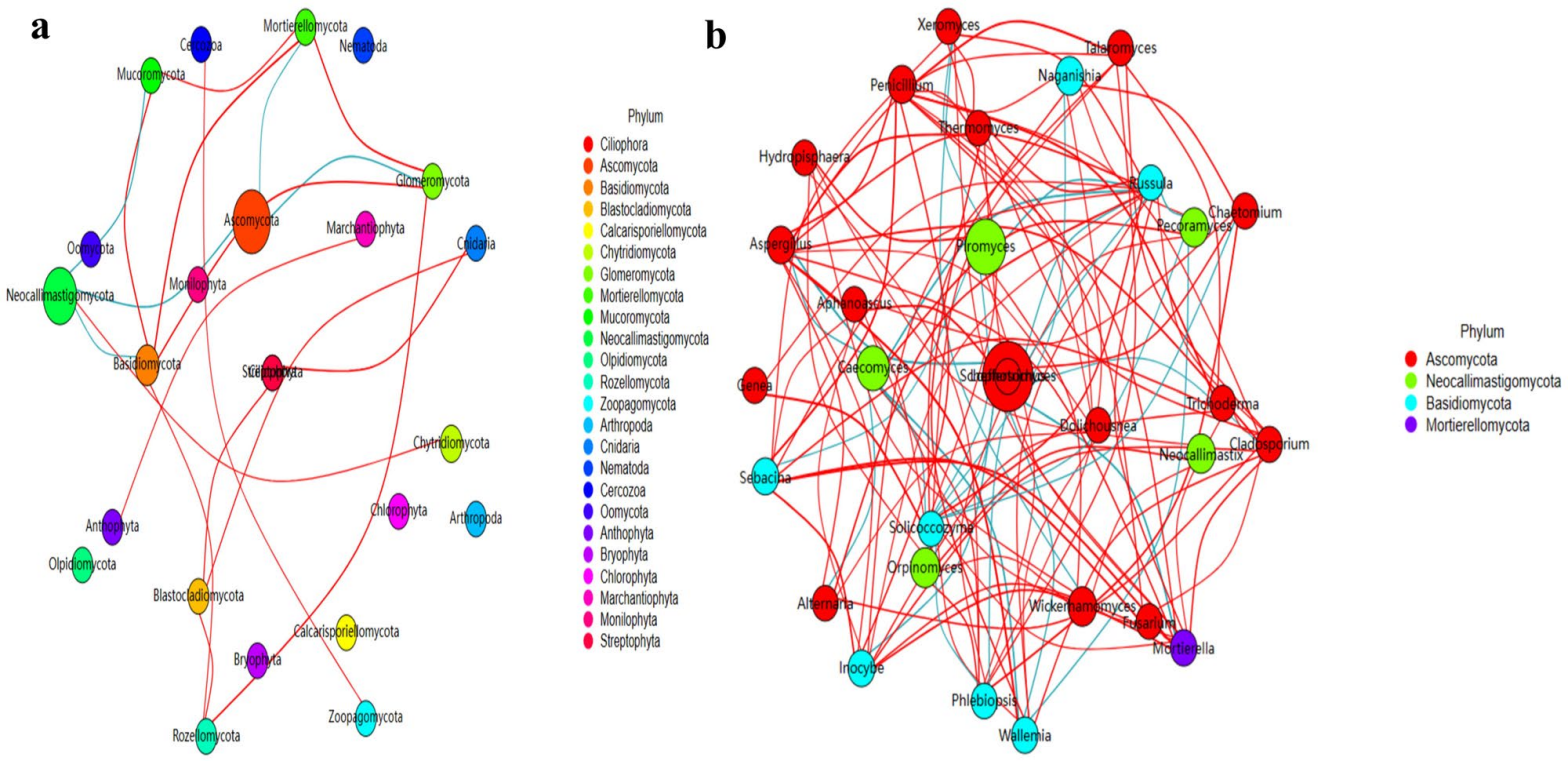
Network analysis of fungi microbiome of SCT sheep. a: Phylum, b: Genera.

No strong difference regarding group distance among SCT groups was found by analysis of NMDS (Figure 6(a)), PCA (Figure 6(b)), PCoA (Figure 6(c)) and Qiime2β (Figure 6(d)); however, effective difference was discovered in plots between AH and BH (*p* < 0.01) and CH (*p* < 0.01) respectively (Figure 6(e)). The LEfSe showed that p__Neocallimastigomycota (*p* < 0.05) and p__Ascomycota (*p* < 0.05) were expressively higher in BH and DH, respectively (Figure 7(a)). At the level of genera, g__*Stephanonectria* (*p* < 0.01) and f__*Hypocreales_fam_Incertae_sedis* (*p* < 0.05) in AH, g__*Myrothecium* (*p* < 0.05), f__*Neocallimastigaceae* (*p* < 0.05), f__*Stachybotryaceae* (*p* < 0.05), g__*Phlebiopsis* (*p* < 0.05), c__*Ustilaginomycetes* (*p* < 0.05), f__*Ustilaginaceae* (*p* < 0.05), c__*Neocallimastigomycetes* (*p* < 0.05), g__*Sporisorium* (*p* < 0.05), o__*Polyporales* (*p* < 0.05), o__*Neocallimastigales* (*p* < 0.05), p__*Neocallimastigomycota* (*p* < 0.05), f__*Phanerochaetaceae* (*p* < 0.05), g__*Piromyces* (*p* < 0.05) and o__*Ustilaginales* (*p* < 0.05) in BH, g__*Simplicillium* (*p* < 0.01), g__*Lecanicillium* (*p* < 0.05), g__*Galerella* (*p* < 0.01), o__*Sordariomycetes_ord_Incertae_sedis* (*p* < 0.05), g__*Conioscypha* (*p* < 0.01), f__*Chaetomiaceae* (*p* < 0.05), c__*Sordariomycetes* (*p* < 0.05), g__*Lophotrichus* (*p* < 0.01), g__*Caecomyces* (*p* < 0.05), o__*Microascales* (*p* < 0.05), g__*Wongia* (*p* < 0.05), g__*Aphanoascus* (*p* < 0.01), f__*Onygenaceae* (*p* < 0.01), o__*Sordariales* (*p* < 0.05), f__*Nectriaceae* (*p* < 0.05), g__*Naganishia* (*p* < 0.05), o__*Filobasidiales* (*p* < 0.05), f__*Filobasidiaceae* (*p* < 0.05), c__*Tremellomycetes* (*p* < 0.05), f__*Bolbitiaceae* (*p* < 0.05), f__*Microascaceae* (*p* < 0.05) and f__*Papulosaceae* (*p* < 0.05) in CH, p__*Ascomycota* (*p* < 0.05) in DH were observed, respectively (Figure 7(b)). The DESeq2 Volcano map exhibits that compared with AH abundance of Ascomycota (*p* < 0.05) and Mucoromycota (*p* < 0.05) were significantly lower in BH and Sebacinales (*p* < 0.05) was significantly lower in DH, respectively (Figure 8(a)). Regarding genus comparison in AH the abundance of *Hydropisphaera* (*p* < 0.001), *Aphanoascus* (*p* < 0.01), *Lophotrichus* (*p* < 0.05) and *Dokmaia* (*p* < 0.05) were significantly higher, while *Occultifur* (*p* < 0.001), *Sarocladium* (*p* < 0.01), *Kazachstania* (*p* < 0.01), *Sebacina* (*p* < 0.01), *Nigrospora* (*p* < 0.01), *Inocybe* (*p* < 0.01), *Meyerozyma* (*p* < 0.05), *Filobasidium* (*p* < 0.05), *Pecoramyces* (*p* < 0.05) and *Gibberella* were remarkably lower. The abundance of *Lophotrichus* (*p* < 0.0001), *Aphanoascus* (*p* < 0.0001), *Chrysosporium* (*p* < 0.001), *Plenodomus* (*p* < 0.01), *Scopulariopsis* (*p* < 0.01), *Acrocalymma* (*p* < 0.01), *Dokmaia* (*p* < 0.01), *Aplosporella* (*p* < 0.01), *Mucor* (*p* < 0.05) and *Scytalidium* were meaningfully higher in CH, while *Occultifur* (*p* < 0.0001), *Stephanonectria* (*p* < 0.0001), *Coprinellus* (*p* < 0.001), *Stachybotrys* (*p* < 0.01), *Fusariella* (*p* < 0.01), *Symmetrospora* (*p* < 0.01), *Filobasidium* (*p* < 0.01), *Pleospora* (*p* < 0.01), *Candida* (*p* < 0.01), *Trichothecium* (*p* < 0.01), *Sarocladium* (*p* < 0.01), *Acrostalagmus* (*p* < 0.01), *Gibberella* (*p* < 0.01), *Inocyb* (*p* < 0.05), *Aureobasidium* (*p* < 0.05), *Kazachstania* (*p* < 0.05) and *Sporisorium* were significantly lower. The abundance of *Xeromyces* (*p* < 0.0001), *Dokmaia* (*p* < 0.05), *Lophotrichus* (*p* < 0.05), *Thermomyces* (*p* < 0.05) and *Aphanoascus* (*p* < 0.05) were expressively higher in DH, while *Inocybe* (*p* < 0.01), *Wickerhamomyces* (*p* < 0.01), *Sebacina* (*p* < 0.01), *Pichia* (*p* < 0.05), *Trichothecium* (*p* < 0.05), *Coprinellus* (*p* < 0.05), *Acrostalagmus* (*p* < 0.05), *Stephanonectria* (*p* < 0.05), *Occultifur* (*p* < 0.05), *Sarocladium* (*p* < 0.05), *Periconia* (*p* < 0.05), *Stachybotrys* (*p* < 0.05), *Candida* (*p* < 0.05), *Fusariella* (*p* < 0.05) and *Microascus* (*p* < 0.05) were significantly lower. The abundance of *Aphanoascus* (*p* < 0.001), *Cystofilobasidium* (*p* < 0.001), *Xeromyces* (*p* < 0.001), *Phoma* (*p* < 0.01), *Rhodotorul* (*p* < 0.01), *Wickerhamomyces* (*p* < 0.01), *Penicillium* (*p* < 0.05), *Ascochyta* (*p* < 0.05), *Trichoderma* (*p* < 0.05) and *Pichia* (*p* < 0.05) were expressively higher in EH. While *Stephanonectria* (*p* < 0.001), *Coprinellus* (*p* < 0.001), *Sarocladium* (*p* < 0.01), *Periconia* (*p* < 0.01), *Trichothecium* (*p* < 0.01), *Papiliotrema* (*p* < 0.01), *Occultifur* (*p* < 0.01), *Pleospora* (*p* < 0.05) and *Simplicillium* (*p* < 0.05) were significantly lower (Figure 8(b)). Network analysis shows that phyla of Ascomycota and Mucoromycota were negatively related to the fungal microbiota of SCT sheep while, Basidiomycota, Blastocladiomycota, Chytridiomycota, Glomeromycota, Mortierellomycota, Zoopagomycota, Cnidaria and Anthophyta were positively related (Figure 9(a)). Genus of *Scheffersomyces*, *Caecomyces*, *Pecoramyces*, *Naganishia* and *Phlebiopsis* were negatively related to the microbiota, while *Piromyces*, *Orpinomyces*, *Wickerhamomyces*, *Neocallimastix*, *Penicillium*, *Aspergillus*, *Sebacina*, *Inocybe*, *Wallemia*, *Mortierella*, C*ladosporium*, *Chaetomium*, *Talaromyces*, *Xeromyces*, *Hydropisphaera*, *Genea*, *Alternaria*, *Russula*, *Thermomyces* and *Aphanoascus* were positively related (Figure 9(b)).

### Function prediction analysis of fungi microbiota in SCT sheep

**Figure 10.**
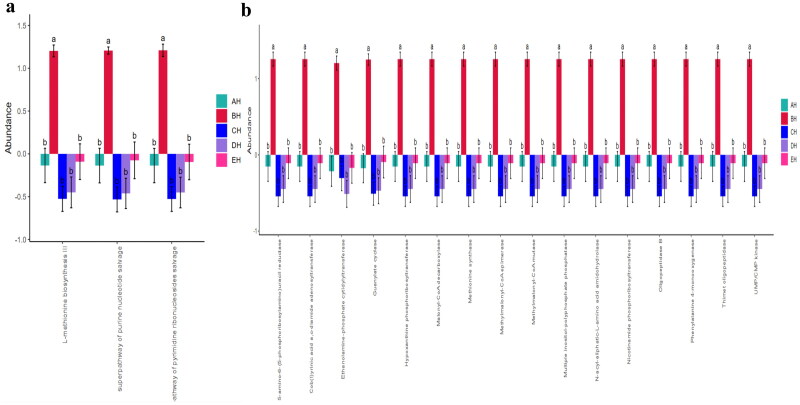
Comparing fungi microbiota function of metaCys pathways (a) and enzyme abundance (b) in SCT sheep.

Fungi microbiota function prediction shows that L-methionine biosynthesis III, super pathway of purine nucleotide salvage and super pathway of pyrimidine ribonucleosides salvage were significantly different among the SCT sheep groups (Figure 10(a)). A total of 16 different enzymes were found significantly different among the five SCT sheep groups (Figure 10(b)).

## Discussion

Currently, researchers are paying attention to fungi microbiota due to their vital functions related to the health of their hosts.^26^ Microbiome analysis is being widely used in forensic investigations,^27^ diseases prediction^28^ and epidemiological studies.^29^

In this study we performed fungi microbiome analysis of SCT sheep with different feeds, and a total of 3,171,271 and 2,719,649 raw and filtered sequences were achieved in SCT sheep. The 111 shared ASVs were found in sheep and it was found that EH (268) had more shared ASVs compared to AH while in comparison with the same DH had less ASVs (171). There was no significant different alpha diversity index in present SCT sheep, which was in line with sheep in different feeding methods;^30^ however, the results of the current study are not in agreement with dairy cattle fed with different dose of cellulose.^31^ The primary phylum found in different sheep groups was Ascomycota in AH (44.64%), DH (54.29%) and EH (52.20%), and Neocallimastigomycota in BH (49.01%) and CH (35.81), respectively. These results are contradictory to the findings in citizen suffering inflammatory bowel disease and paediatric inhabitants,^32^^,^^33^ in which Ascomycota was found dominant phylum. Different results were found at genera level, *Scheffersomyces* was found main genera in AH (29.09%), CH (15.21%), DH (38.76%) and EH (18.37%), while *Piromyces* (31.97%) was the dominated genera in BH. Besides shifts of fungi structure in different taxa difference of feed also changed the composition of fungi microbiota in SCT sheep which was confirmed by grouping and clustering heat map along with evolutionary tree of species with heat map. The changed fungi microbiota affected functions of metaCys pathways and enzymes in SCT sheep.

Distinguished fungi species in SCT sheep were explored through LEfSe and it was discovered that p__Neocallimastigomycota and p__Ascomycota were higher in BH and DH respectively. Two genera in AH, 14 in BH, 22 in CH and 1 in DH were significantly higher, which proved altering of microbiota in SCT animals. For identifying different marker fungi species in sheep of the current study DESeq2 volcano map analysis was used which detected 3 phyla and 47 genera. Among those genus *Sarocladium* was discovered to be a pathogenic fungus.^34^ The lower abundance of those genera in BH, CH and EH indicates that concentrated feed decreases fungal infection in SCT sheep. *Kazachstania* is a pathogen commonly reported in veterinary practice,^35^ lower abundance of this genus in BH and DH shows that concentrated feed II can be used limit the growth of the aforementioned pathogen in sheep. Higher abundance of *Gibberella* was reported in Crohn’s disease.^36^ The lower abundance of *Gibberella* in BH and CH suggests that concentrated feed I decreased the said hazardous fungi in SCT sheep. Lower abundance of *Scytalidium* was reported in ulcerative colitis,^37^ more abundant of this genus in CH indicates that concentrated feed I promotes the colonization of *Scytalidium* in sheep. Previously, it has been reported that *Coprinellus* was found to be dominating in *Cryptosporidium parvum* infected yaks.^38^ Lower abundance of this genera in CH, DH and EH demonstrates that concentrated feeds relieve the diarrhea in ruminants. The higher abundance of *Symmetrospora*, *Filobasidium* and *Aureobasidium* was reported in *C. parvum* infected yaks,^38^ UC patients^39^ and newborns with bronchopulmonary dysplasia damage.^40^ This confirms that concentrated feed I decrease pathogenic fungi genera in sheep. Species of *Candida* and *Wickerhamomyces* are to be opportunistic pathogens^37^^,^^41^ lower abundance of *Candida* in CH and DH along with *Wickerhamomyces* in DH and EH revealed that concentrated feed limited the pathogenic fungal growth in gut of sheep. Previous studies found higher abundance of *Pichia* in people with alcohol related liver damages,^42^ lower abundance of this genus in DH demonstrates that concentrated feed II recover liver injury. It is reported that lower abundance of *Penicillium* in mice treated with fluoride,^43^ more abundant of this genus in EH shows that concentrated feed# 3 promotes the these fungal in SCT sheep. *Aphanoascus*, *Dokmaia*, *Occultifur*, *Sebacina*, *Stachybotrys* and *Stephanonectria* are environmental genera,^44–49^ which may have little relationship with the fungi microbiota in SCT sheep.

## Conclusion

Concentrate feeding regulates the gut fungal biome of SCT sheep by enhancing the abundance of positive genera like *Scytalidium* and decreasing negative pathogenic fungal strains which include *Sarocladium*, *Kazachstania*, *Gibberella*, *Scytalidium*, *Candida* and *Wickerhamomyce*. These findings can be critical related to efficient feeding for Suffolk cross to Tibetan sheep on cold plateaus. Therefore, further studies need to conduct to related the results of this study for efficient feeding of Suffolk cross to Tibetan sheep on the plateaus.

## Data Availability

All raw sequences data were deposited in the NCBI Sequence Read Archive database under the following accession number: PRJNA1013103.
